# Accelerated Bone Mass Senescence After Hematopoietic Stem Cell Transplantation

**Published:** 2013-01-04

**Authors:** B Serio, L Pezzullo, R Fontana, S Annunziata, R Rosamilio, M Sessa, V Giudice, I Ferrara, M Rocco, G De Rosa, P Ricci, L Tauchmanovà, N Montuori, C. Selleri

**Affiliations:** 1 Hematology and Bone Marrow Transplant Center, Department of Medicine and Surgery, University of Salerno, Italy;; 2 Hematology, Federico II University of Napoli, Italy;; 3 Department of Translational Medicine and Pediatrics, Federico II University of Napoli, Italy (cselleri@unisa.it)

**Keywords:** osteoporosis, hemopoietic stem cell transplantation, bisphosphonates

## Abstract

Osteoporosis and avascular necrosis (AVN) are long-lasting and debilitating complications of hematopoietic stem cell transplantation (HSCT).

We describe the magnitude of bone loss, AVN and impairment in osteogenic cell compartment following autologous (auto) and allogeneic (allo) HSCT, through the retrospective bone damage revaluation of 100 (50 auto- and 50 allo-HSCT) long-term survivors up to 15 years after transplant. Current treatment options for the management of these complications are also outlined.

We found that auto- and allo-HSCT recipients show accelerated bone mineral loss and micro-architectural deterioration during the first years after transplant. Bone mass density (BMD) at the lumbar spine, but not at the femur neck, may improve in some patients after HSCT, suggesting more prolonged bone damage in cortical bone. Phalangeal BMD values remained low for even more years, suggesting persistent bone micro-architectural alterations after transplant. The incidence of AVN was higher in allo-HSCT recipients compared to auto-HSCT recipients. Steroid treatment length, but not its cumulative dose was associated with a higher incidence of bone loss. Allo-HSCT recipients affected by chronic graft versus host disease seem to be at greater risk of continuous bone loss and AVN development. Reduced BMD and higher incidence of AVN was partly related to a reduced regenerating capacity of the normal marrow osteogenic cell compartment.

Our results suggest that all patients after auto-HSCT and allo-HSCT should be evaluated for their bone status and treated with anti-resorptive therapy as soon as abnormalities are detected.

## I. INTRODUCTION

Patients with several malignant and non-malignant hematological diseases such as acute leukemia, especially acute lymphoblastic leukemia in children, lymphoma, myeloma, major thalassemia and severe hemophilia are at risk for bone damage [1–15]. Moreover, various hematological related therapies, including chemotherapy, glucocorticoids, hormonal agents and newer targeted therapies may affect bone health resulting in long-term skeletal disorders, particularly osteopenia, osteoporosis, fractures and avascular necrosis (AVN) [16–23].

In the last four decades the number of autologous (auto) and allogeneic (allo) hematopoietic stem cell transplantation (HSCT) in the treatment of malignant and non-malignant hematological diseases has grown together with a progressive increase of long-term survivors due to a decrease of transplant-related mortality.

With the number of long-term survivors after HSCT progressively increasing, early and late complications of this procedure have gained much more attention. Osteoporosis, fractures and AVN leading to pain and disability represent some of more frequent complications which may worsen the quality of life of long-term survivors after HSCT [24].

The aim of this review is to describe the magnitude of bone loss and AVN following HSCT and their current management, as well as to examine the role of marrow microenvironment in the development of bone loss after HSCT.

## II. METHODOLOGY

### Subjects

A retrospective cohort of 100 patients who had undergone auto- (n=50) or allo-HSCT (n=50) for a hematological malignancy, and had survived free of disease for one or more years post-HSCT, was evaluated in this study. Clinical features of the patients and underlying diseases are summarized in Table 1. Their age at transplantation ranged between 20 and 52 years (median, 32) and their post-HSCT follow-up lasted from 1 to 15 years (median, 6). Primary diseases were acute (n=44) or chronic (n=13) myeloid leukemia, Hodgkin disease (n=17), non-Hodgkin lymphoma (n=12), and multiple myeloma (n=14). Auto- and allo-HSCT groups were similar for age, body mass index (BMI), and time elapsed from transplant.

### Measurement of bone mass density

Bone mass density (BMD) was measured by dual energy X-ray absorptiometry (DEXA) at lumbar spine (L1–L4) and femoral neck, and by quantitative phalangeal ultrasonometry (P-QUS), as previously described [25]. BMD was expressed as Z scores, that assess standard deviation (SD) of patients’ BMD compared with normal values from age and sex-matched controls, respectively. Osteopenia and osteoporosis were defined by a Z score below −1 and −2.5 SD, respectively [26, 27].

Diagnosis of AVN suspected cases was documented by magnetic resonance imaging (MRI).

### Measurement of osteogenic precursors

The marrow compartment of osteogenic precursors was evaluated in 35 auto- and allo-HSCT recipients by growing colony-forming units-osteogenic cells (CFU-O), defined by their ability to express alkaline phosphates activity from enriched mesenchymal stem cells, as previously described [28].

### Statistical analysis

Statistical analysis of the data (expressed as mean ± SD and ± SEM as appropriate throughout the text and in figures) was performed using paired students t-tests for non-parametric variables. Statistical significance was considered for p < 0.05.

## III. RESULTS

### Bone mineral density in transplanted patients

**Fig. 1.**
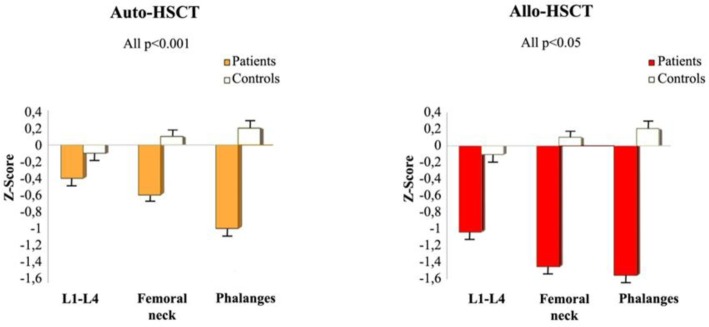
**Bone mass evaluation in transplanted patients versus normal controls.** Mean values of Z score (expressed as mean ± SD) at lumbar spine (L1–L4), femoral neck and phalanges, in allogeneic and autologous hemopoietic stem cell transplantation (auto- and allo-HSCT) recipients. Each column represents the mean values of Z scores, vertical bars represent the standard error of mean.

At the time of testing (mean follow-up after HSCT: 65 months; range: 1–15 years), lumbar spine (Z score mean value: −0.4 and −0.9 in auto- and in allo-HSCT recipients, respectively; *p*<0.05), femoral neck (−0.6 and −1.4 in auto- and in allo-HSCT recipients, respectively; *p*<0.05) and phalanges (−1 and −1.5 in auto- and in allo-HSCT recipients, respectively; *p*<0.05) BMD were significantly reduced in comparison with BMD of 100 healthy controls (*p*<0.001 in all examined sites) (Figure 1).

By DEXA and P-QUS, we could not document significant correlation between BMD reduction and age at the time of HSCT, cumulative dose of prednisone and cyclosporine A, length of steroid treatment and follow-up after BMT (Table 1). Development of acute and chronic graft versus-host disease (GVHD), requiring long lasting high dose steroid treatment, was associated with a more severe BMD reduction in all bone sites (<0.05).

Auto- and allo- HSCT recipients who were evaluated > 3 years after transplant had significantly higher (p< 0.001) lumbar BMD than patients evaluated < 3 years. On the other hand, no difference was seen in HSCT recipients evaluated before or after 3 years since transplant at the femoral neck and phalanges (data not shown). Phalangeal BMD values remained low even after more than 6 years (data not shown).

### Avascular necrosis in transplanted patients

Eight patients developed AVN, 1 to 15 years (median, 28) following HSCT: 6 (12%) after allo-HSCT and 2 (4%) after auto-HSCT (*p*<0.05). A total of 15 joints were affected and all patients had femoral head involvement, which required surgical management in 4 of them. All but one patient affected by AVN in the allogeneic setting were suffering from chronic extensive GVHD; its exacerbation or recurrence was documented in 3/6 patients shortly (1–6 months) before the AVN diagnosis. Concerning possible risk factors, AVN was related to allogeneic type of transplant, chronic extensive GVHD, longer steroid treatment and higher cumulative steroid dose (data not shown).

### Osteogenic compartment in transplanted patients

**Fig. 2.**
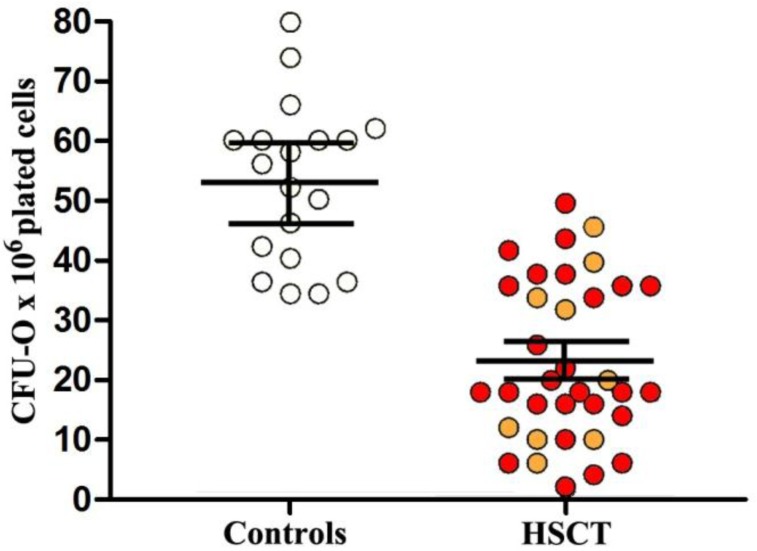
**Number of colony forming units-osteogenic progenitors (CFU-O) in hematopoietic stem cell transplantation (HSCT) recipients and normal controls.** Each orange and red dot represents an auto- and allo-HSCT recipient. Horizontal bars represent mean values, vertical bars represent the standard error of mean.

The marrow compartment of osteogenic precursors, measured as CFU-O cells, was decreased 2 to 3- fold in auto- and allo-transplanted recipients compared to normal donors (30±6 and 21±5 in auto- and allo-HSCT recipients, respectively, vs 55±4/10^5^ cells plated in normal controls; all *p*<0.001) (Figure 2). Analyzing the effect of the time elapsed since HSCT on marrow CFU-O numbers, we found the marrow CFU-O compartment markedly depleted during the first years after transplant (data not shown). Between 6 and 15 years, the mean marrow CFU-O cell number tended to increase (13 ±3 vs 33 ±5 before and after 3 years, respectively; *p*<0.05), although most patients showed a number of CFU-O permanently below that observed in normal controls. CFU-O growth correlated significantly with lumbar, femoral and phalangeal bone loss (all *p*<0.01). Chronic GVHD was correlated with a lower number of CFU-O colonies *in vitro* (*p*<0.01).

**Fig. 3.**
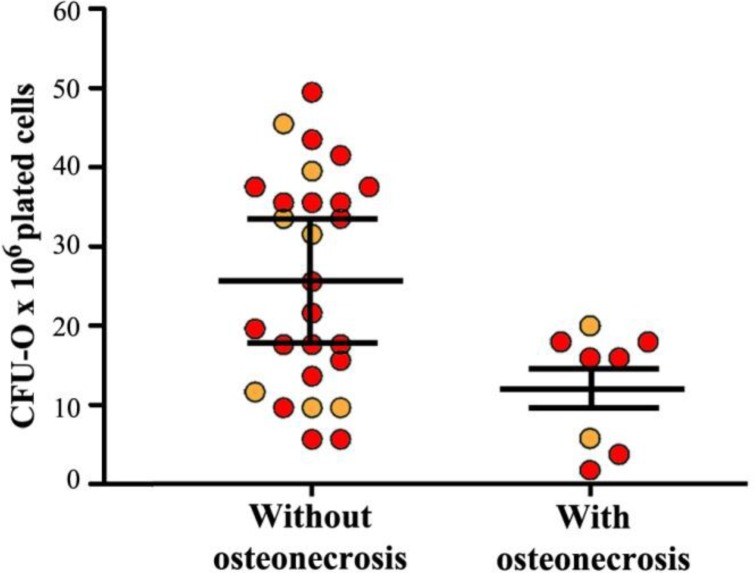
**Number of colony-forming units-osteogeneic progenitors (CFU-O) in auto- (orange dot) and allo (red dot) hemopoietic stem cell transplantation (HSCT) recipients with and without osteonecrosis.** Each dot represents a subject studied. Horizontal bars represent mean values, vertical bars represent the standard error of mean.

Finally, almost all transplanted patients who had AVN showed a number of CFU-O below that observed in transplanted patients without this complication (CFU-O: 24.5±3 vs 12.4±4 in HSCT patients without and with osteonecrosis, respectively; *p*<0.05) (Figure 3).

## IV. DISCUSSION

Osteopenia and osteoporosis are relatively common early complications of auto- and allo-HSCT. They are attributable to the influence of multiple factors including myeloablative conditioning regimens, huge cytokine release at the time of transplant, altered kidney, liver and bowel function resulting in reduced intake and altered metabolism of calcium and vitamin D, frequent gonadal failure and, in allogeneic HSCT setting, long-lasting high-dose steroids and cyclosporin-A [29, 30]. In the current study, we retrospectively followed 100 consecutive patients who had undergone auto- and allo-HSCT and survived one or more years. As already suggested by our and other studies, we documented a marked decrease in BMD after auto- and allo-HSCT both at the lumbar spine (25%) and even more at the femoral neck (50%). We have confirmed that a significant decrease in BMD at lumbar and femoral neck level appears early after transplant and seems to continue over the first 3 years with no further deterioration afterwards.

Although bone density is among the strongest predictors of the mechanical behavior of trabecular bone, the whole bone strength is determined also by bone quality. Apart from bone mineralization, bone architecture, turnover, and damage accumulation also account for bone quality. Several data show that trabecular micro-architecture influences trabecular bone strength. Early bone loss may consist of both demineralization and architectural damage with associated organic matrix deficit. DEXA measures bone density and mineralization but does not provide information on architectural damage and bone formation [31]. High-resolution computed tomography and MRI allow for three-dimensional assessment of trabecular structure, but their use in the routine clinical practice for diagnosis and follow-up of bone damage is limited, being too costly and time-consuming. Ultrasonographic evaluation by phalangeal QUS is a safe and less expensive procedure, which permits to assess more physical properties of bone tissue and to account for more structural changes than DEXA [32]. Phalangeal QUS allows to evaluate bone density and elasticity, trabecular orientation and cortical-to-trabecular ratio, all of which are influenced by mineral content and organic matrix. In HSCT recipients, we have further confirmed by DEXA that, while mineralization seems to improve at trabecular rich sites such as lumbar spine, no improvement was detected at cortical bone such as femoral neck; in addition, no improvement was revealed by phalangeal QUS even several year after transplant.

Allo- and auto-HSCT recipients were mostly pooled together in several clinical studies on bone complications after HSCT, whereas there are considerable differences between these two settings. The differences consist in a higher grade of immunologic derangement and more prolonged use of immunosuppressive treatments needed to avoid development of acute and chronic GVHD in the al-logeneic setting [27]. Apart from women which may experience ovarian failure after auto- and allo-HSCT, in the setting of allogeneic HSCT, we have also documented that an important high risk factor is the development of chronic GVHD requiring prolonged high-dose of steroids [33–37].

Avascular necrosis (AVN) has been described in 3–41% of patients who had received an organ transplant, with femoral head being the prevalent localization [38]. AVN is the result of multiple triggering factors such as metabolic disorders, local vascular damage with transient or permanent loss of blood supply to the bone, increased intra-osseous pressure and mechanical stress leading to demineralization, and death of trabecular bone and collapse.

The process is mostly progressive, resulting in joint destruction within three to five years if left untreated. AVN occurred in 8 of 100 long-term survivors (8%), within 3–15 years (median, 3.6) after-HSCT, all but two of them having received an allo-HSCT. Surgical treatment was required in half of HSCT patients because of functional limitations. A significant statistical association was found between AVN occurrence, allogeneic type of transplant, presence and grade of GVHD, steroid treatment length, and cumulative dose [17, 39]. In addition, we have recently reported a close relationship between AVN occurrence and flare up of chronic GVHD [38].

**Fig. 4.**
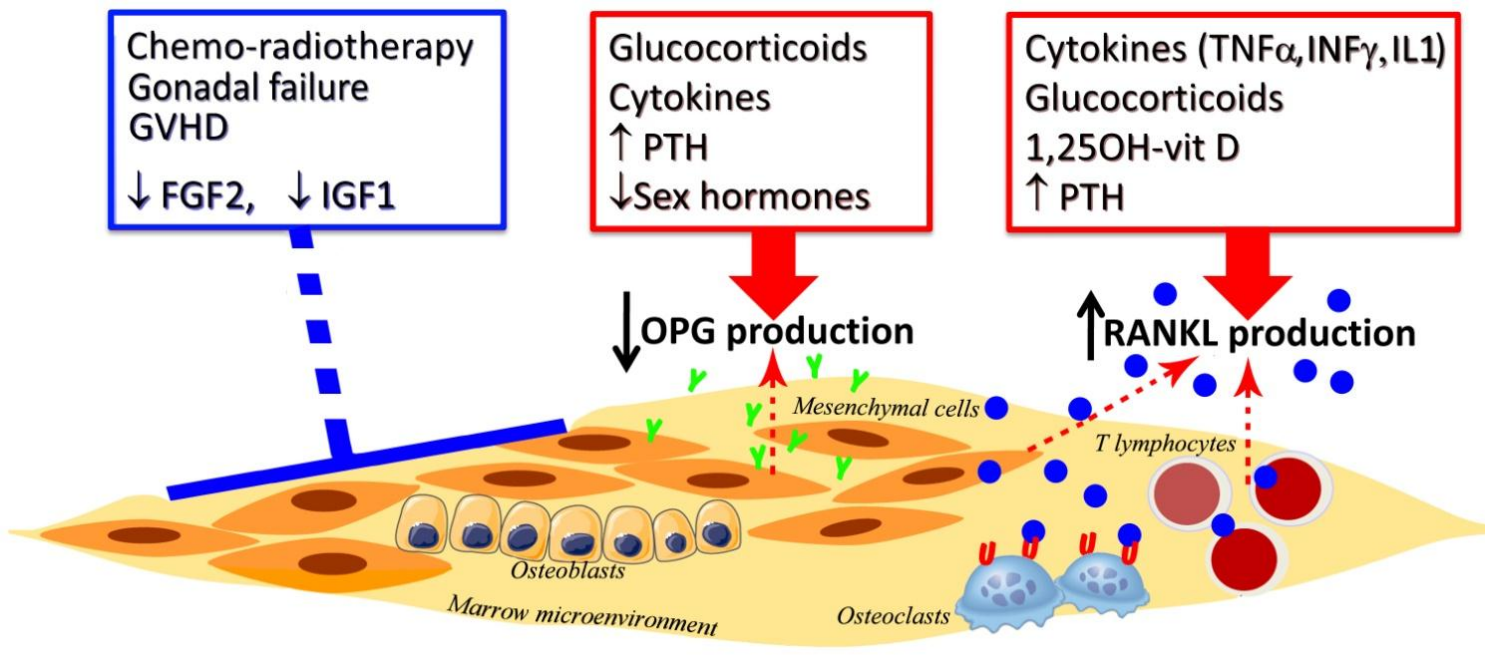
**Critical factors involved in bone remodeling after hemopoietic stem cell transplantation.** Chemotherapy, radiotherapy, gonadal failure, acute and chronic graft versus host disease (GVHD) and growth factors (such as fibroblast growth factor-2 -FGF2-, insulin growth factor-1 -IGF1-) may affect directly bone marrow microenvironment altering the capacity to synthesize osteoprotegerin (OPG) and receptor activator for nuclear kappa-B ligand (RANKL). In particular, high dose of steroids, cytokines, high levels of parathyroid hormone (PTH) and low levels of sexual hormones decrease OPG production by osteoblastic progenitors, whereas pro-inflammatory cytokines (such as tumor-necrosis factor-α -TNFα-, interleukin-1 -IL1- and interferon-γ -IFNγ -), long-lasting glucocorticoids, 1,25OH-vitamin D and PTH increase RANKL production by osteoblasts and T lymphocytes. All of these marrow microenvironment changes may increase RANKL/OPG ratio stimulating osteoclastic activity and bone resorption. Symbols: **Y**, OPG; blue **O**, RANKL; **U**, RANK.

Bone remodeling, specifically the balance between formation and resorption, is the biologic process that mediates architectural changes influencing the whole bone strength. We have documented a marked and permanent quantitative and qualitative defect in the marrow osteogenic cell compartment, suggesting that inability to restore a normal number of osteoblastic precursors in the bone microenvironment may account at least in part for severe bone damage after auto- and allo-HSCT. The reduced repopulating capacity of osteoblast precursors after HSCT is likely related to the effects of chemotherapy/radiotherapy, to the concomitant endocrine disorders, immunosuppressive treatments, and alteration in the balance of cytokines and growth factors. According to the above described long-lasting deficit of osteogenic progenitors, we have recently documented within the bone marrow microenvironment of these HSCT recipients low osteoprotegerin (OPG) levels, exacerbated by a high ratio for receptor activator for nuclear kappa B ligand (RANKL)/OPG favouring bone resorption after HSCT (Figure 4) [40].

Few data are available so far on the treatment of bone loss in HSCT recipients. Early diagnosis of osteoporosis or early bone senescence in this particular population is a major aim to promptly start appropriate supportive measures, such as lifestyle modification, calcium and vitamin D supplementation or bisphosphonates. However, there is sufficient evidence that common preventive measures for bone loss, such as calcium and vitamin D supplements and sex steroid replacement, are ineffective in HSCT recipients. Bisphosphonates are currently the most effective inhibitors of osteoclastic bone resorption; they directly impair the ability of osteoclasts to adhere to the bone surface, and inhibit osteoclast activity by decreasing osteoclast progenitor development and recruitment and by promoting osteoclast apoptosis. In addition, recent evidences have been accumulated on the anabolic bone effects of bisphosphonates, enhancing osteoblast proliferation, and preventing osteoblast apoptosis [41–43]. Oral bisphosphonates, widely used for treating osteoporosis, have been shown to improve bone BMD and decrease the rate of fractures in various patient populations [44]. However, the use of these drugs in the clinical routine of HSCT recipients is limited by their poor gastrointestinal tolerance, variable oral bioavailability and long-term compliance. We have previously reported that zoledronic acid, given as three consecutive monthly doses of 4 mg each every years, is able to obtain a rapid and measurable increase of bone mass. Given the persistent bone loss at trabecular and cortical rich skeletal sites in HSCT recipients, zoledronic acid may be considered the treatment of choice in the near-transplant period for the prevention of bone loss. We also documented that beneficial effects on lumbar and femoral BMD of zoledronic acid treatment in HSCT patients were associated with a significant improvement of the osteogenic progenitors [28]. In addition, we have recently documented in a small cohort of HSCT recipients that 1-year treatment with risedronate increases lumbar spine BMD and prevents bone loss at the femoral neck [44]. Clinical experience with the human monoclonal antibody anti-RANKL (denosumab), which is documented to be effective in non meta-static prostate and breast cancer as well as in postmenopausal osteoporosis, as well as with the parathyroid hormone derived peptides, with hormone analogues and selective estrogen receptor modulators, is still limited in HSCT recipients [30, 45, 46].

## V. CONCLUSIONS

In this study, we have confirmed that an accelerated and persistent multifactorial bone loss occurs in long-term survivors after HSCT, being more severe in the allogeneic setting. Moreover, osteoporosis is more frequent than AVN in patients after allo-HSCT. Several new risk factors have been identified and include a dysregulation of the immune system occurring during acute and chronic GVHD and their treatments, persistently reduced regenerating capacity of normal osteogenic cell compartment, and osteoclastic activation by alterations in the RANKL/OPG balance.

DEXA bone density testing remains an accurate method to detect bone loss in HSCT recipients. Since phalangeal QUS is a safe and easy method, it should be more frequently used for early recognition and monitoring of BMD reduction as well as for decision-making in transplanted patients who may need specific therapy. Oral and parenteral bisphosphonates are currently the only treatments able to prevent and treat accelerated bone mass senescence after transplant. The choice between oral or intravenous bisphosphonate therapy in patients who had undergone HSCT should be made on the basis of individual clinical conditions, including presence, grading and localization of GVHD as well as prevalent site of bone loss. Oral risedronate and intermittent short course of zoledronic acid were easily manageable and effective in increasing densitometric values at trabecular skeletal sites. Zoledronic acid was the only therapy that improved also femoral BMD. At least part of these effects may be related to the bisphosphonates’ ability to partly restore the persistent post-transplant reduction of osteogenic progenitors. Despite the evidence of only partial effectiveness of preventive anti-resorptive treatments, it is reasonable to start administration of these drugs before or at the time of HSCT in all patients, as soon as bone abnormalities are detected, and continue them at least for the first years after transplant.

## Figures and Tables

**TABLE I:** PATIENTS CHARACTERISTICS

	Hematopoietic stem cell transplant		Controls

	Allogeneic	Autologous	
Number	50	50	100
Age (years)	32±12*	41±12	36±13
Female/Male	23/27	21/29	43/57
Diagnosis:			
AML	30	14	
CML	13	-	
HD	2	15	
NHL	2	10	
MM	3	11	
Follow-up (months)	17.5±17	16.9±11	-
BMI (kg/m^2^)	25±5.7	26±4.8	25.3±5
Conditioning	BUCY2/BEAM/TTCY MEL	BUCY2/BEAM/TTCY MEL	-
Steroid dose (g/m^2^)	7.6±3.4	7.8±3.5	
duration (months)	11.7±9.3°*	6.9±6	-
CsA dose (g/m^2^)	14±10	-	-
duration (months)	8±8	-	-

Abbreviations: AML, Acute Myeloid Leukemia; CML, Chronic Myeloid Leukemia; HD, Hodgkin’ Disease; NHL, Non Hodgkin Lymphoma; MM, Multiple Myeloma; BMI, Body Mass Index; BU-CY2, Busulfan – Cyclophosphamide; BEAM, Carmustine - Etoposide – Cytarabine - Melphalan; TTCY, ThioTepa – Cyclophosphamide; MEL, Melphalan; CsA, Cyclosporine A. Symbols:

°, expressed as prednisone equivalents.;

*, p<0.05 vs autologous stem cell transplantation.
